# AlphaFold predicted structure of the Hsp90-like domains of the neurodegeneration linked protein sacsin reveals key residues for ATPase activity

**DOI:** 10.3389/fmolb.2022.1074714

**Published:** 2023-01-13

**Authors:** Laura Perna, Matteo Castelli, Elena Frasnetti, Lisa E. L. Romano, Giorgio Colombo, Chrisostomos Prodromou, J. Paul Chapple

**Affiliations:** ^1^ William Harvey Research Institute, Faculty of Medicine & Dentistry, Queen Mary University of London, London, United Kingdom; ^2^ Department of Chemistry, University of Pavia, Pavia, Italy; ^3^ Biochemistry and Biomedicine, School of Life Sciences, University of Sussex, Brighton, United Kingdom

**Keywords:** Hsp90, sacsin, ARSACS, neurodegeneration, ataxia, molecular chaperone

## Abstract

The ataxia-linked protein sacsin has three regions of partial homology to Hsp90’s N-terminal ATP binding domain. Although a crystal structure for this Hsp90-like domain has been reported the precise molecular interactions required for ATP-binding and hydrolysis are unclear and it is debatable whether ATP biding is compatible with these domains. Furthermore, the Identification of a sacsin domain(s) equivalent to the middle domain of Hsp90 has been elusive. Here we present the superimposition of an AlphaFold structure of sacsin with yeast Hsp90, which provides novel insights into sacsin’s structure. We identify residues within the sacsin Hsp90-like domains that are required for ATP binding and hydrolysis, including the putative catalytic arginine residues equivalent to that of the Hsp90 middle domain. Importantly, our analysis allows comparison of the Hsp90 middle domain with corresponding sacsin regions and identifies a shorter lid segment, in the sacsin ATP-binding domains, than the one found in the N-terminal domain of Hsp90. Our results show how a realignment of residues in the lid segment of sacsin that are involved in ATP binding can better match equivalent residues seen in Hsp90, which we then corroborated using molecular dynamic simulations. We speculate, from a structural viewpoint, why some ATP competitive inhibitors of Hsp90 may not bind sacsin, while others would. Together our analysis supports the hypothesis that sacsin’s function is ATP-driven and would be consistent with it having a role as a super molecular chaperone. We propose that the SR1 regions of sacsin be renamed as HSP-NRD (Hsp90 N-Terminal Repeat Domain; residues 84-324) and the fragment immediately after as HSP-MRD (Hsp90 Middle Repeat Domain; residues 325-518).

## Introduction

Mutations which lead to loss of function of the protein sacsin cause the neurodegenerative disorder Autosomal Recessive Spastic Ataxia of Charlevoix Saguenay or ARSACS (Engert et al., 2000; Fogel and Perlman, 2007; Martin et al., 2007). Although a very rare disease, ARSACS is thought to be the second most common form of autosomal recessive cerebellar ataxia after Friedrich’s ataxia (Fogel and Perlman, 2007). It normally manifests in childhood and is characterised by progressive cerebellar ataxia, peripheral neuropathy, and spasticity.

**FIGURE 1:**
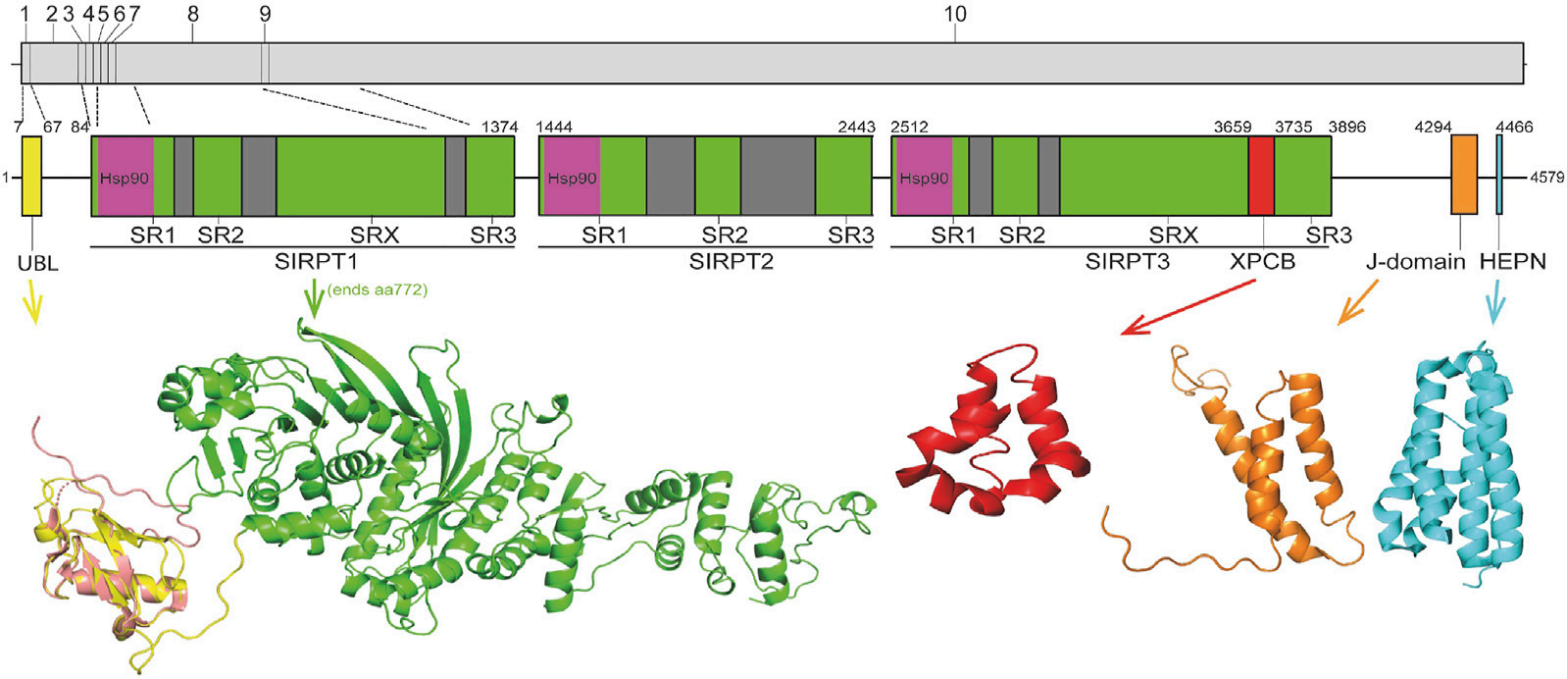
Schematic representation of sacsin domain structure. Regions of sacsin with homology to domains of other proteins are indicated (numbers correspond to amino acid position) with domains where structure has been solved or modelled shown (UBL, Hsp90, XPCB, J-domain and HEPN). The internal repeats of sacsin are also indicated and the structure of the sacsin transcript is also shown (with exons numbered and dotted lines indicating exons encoding different regions of sacsin). The crystal structure of the sacsin UBL (PDB 5VSX; salmon) is superimposed on the AlphaFold predicted UBL structure (yellow).

Sacsin is an extremely large (4,579 amino acid) multidomain protein that is conserved through vertebrate evolution (Romano et al., 2013). There is no overall structural similarity between sacsin and other proteins, however, it does contain conserved domains (Figure 1). Specifically, from the N- to C-terminus sacsin incorporates; 1) a ubiquitin-like domain (UBL) that interacts with the 19S cap of the 26S proteasome and mediates protein degradation (Parfitt et al., 2009; Menade et al., 2018); 2) three supra domains known as sacsin internal repeats (SIRPT), that can be further divided into smaller sub-repeats known as SR1, SR2, SR3 and SX (SIRPT2 lacks the SRX repeat), with each SR1 containing a region of homology to the Hsp90 N-terminal ATPase domain (Anderson et al., 2010; Romano et al., 2013); 3) a xeroderma pigmentosum complementation group C binding (XPCB) domain that interacts with the ubiquitin ligase and Angelman syndrome protein Ube3A (Kamionka and Feigon, 2004; Greer et al., 2010); 4) a J-domain that binds and activates Hsp70 (Parfitt et al., 2009; Anderson et al., 2010); and, 5) a higher eukaryotes and prokaryotes nucleotide-binding domain (HEPN) that may promote sacsin dimerization (Grynberg et al., 2003; Girard et al., 2012a).

The domain structure of sacsin provides a link to both the ubiquitin proteasome system and molecular chaperones, suggesting a potential function in protein quality control systems. Although there is some evidence supporting this (Parfitt et al., 2009; Gentil et al., 2019), the precise role of sasin is unknown and it is unclear why its loss results in a complex cellular phenotype that includes mitochondrial dysfunction (Girard et al., 2012a; Bradshaw et al., 2016; Morani et al., 2022), altered intermediate filament cytoskeleton organisation (Lariviere et al., 2015; Duncan et al., 2017; Gentil et al., 2019), altered microtubule dynamics (Francis et al., 2022) and disrupted intracellular trafficking (Romano et al., 2022).

One possibility is that sacsin functions directly as a super molecular chaperone and if this is the case its three regions of homology to Hsp90 (in the SR sub-repeats of SIRPT1, 2 and 3) could indicate its action, like Hsp90, is driven by ATP binding and hydrolysis. Currently, sequence alignment and crystallographic analysis have shown that the sacsin SR1 has structural similarity to the Bergerat protein fold of Hsp90, which forms a nucleotide binding pocket from a sandwich of four β-sheets and two α-helices (Menade et al., 2018). However, the current understanding is that sacsin’s SR1 Bergerat fold is not entirely structurally conserved with that of Hsp90 proteins but does show some conservation of amino acids important for ATP binding and hydrolysis. It is not clear whether SR1 has a segment of structure that could act as an ATP-binding lid, which may be consistent with recombinant SIRT1-SR1 having low levels of ATPase activity in a steady state assay (Menade et al., 2018). In another study where a larger domain of mouse sacsin was used, from the N-terminus to the start of SIRT2, ATPase activity equivalent to yeast Hsp90 was demonstrated (Anderson et al., 2010). These data may be consistent with a region of sacsin outside of the SR1 domain contributing to its ATPase activity. It is also interesting to note that key residues in the SR1 putative nucleotide binding pocket (excluding the lid segment) are completely conserved between the SIRT domains of sacsin, suggesting they are important for function (Menade et al., 2018). Moreover, there is evidence for the ATPase function of the SR1 being important for sacsin function, as it has been shown that the ARSACS mutation D169Y suppresses sacsin activity (Anderson et al., 2010). Importantly, Hsp90 possesses a putative catalytic-loop arginine, found in its middle-domain that is required to complete the ATPase unit of Hsp90 and thus allow hydrolysis of ATP. However, at first sight sacsin appears to have no obvious domains equivalent to the middle domain of Hsp90 and the putative catalytic arginine residues have not been identified. Thus, it not clear if sacsin has a domain equivalent to the middle domain of Hsp90, which would provide the critical function for efficient ATP hydrolysis. Conformation of Hsp90-like function would allow us to better understand the molecular chaperone role of sacsin.

To resolve this controversy and better understand the molecular function of sacsin we hypothesised that recent advances in protein structural modelling could be exploited to identify key residues within sacsin that could mediate ATP binding and catalysis.

### Findings from the superimposition of yeast Hsp90 and the AlphaFold model of sacsin

We hypothesis that sacsin is an ATP-dependent super molecular chaperone. This theory is based on an analysis showing that the structure of sacsin is compatible with the known mechanism for ATPase hydrolysis by Hsp90, although this may not extend to dimerization of the ATP binding domains of sacsin, as observed with Hsp90. We propose that a unique piece of structure, as part of the HSP-N-terminal Repeat Domain (HSP-NRD), not found in the middle domain of Hsp90, may provide stability for closure of the lid over bound ATP.

### AlphaFold predicted sacsin SR domains are structurally consistent with the ATPase constraints of the Begarat fold

**FIGURE 2:**
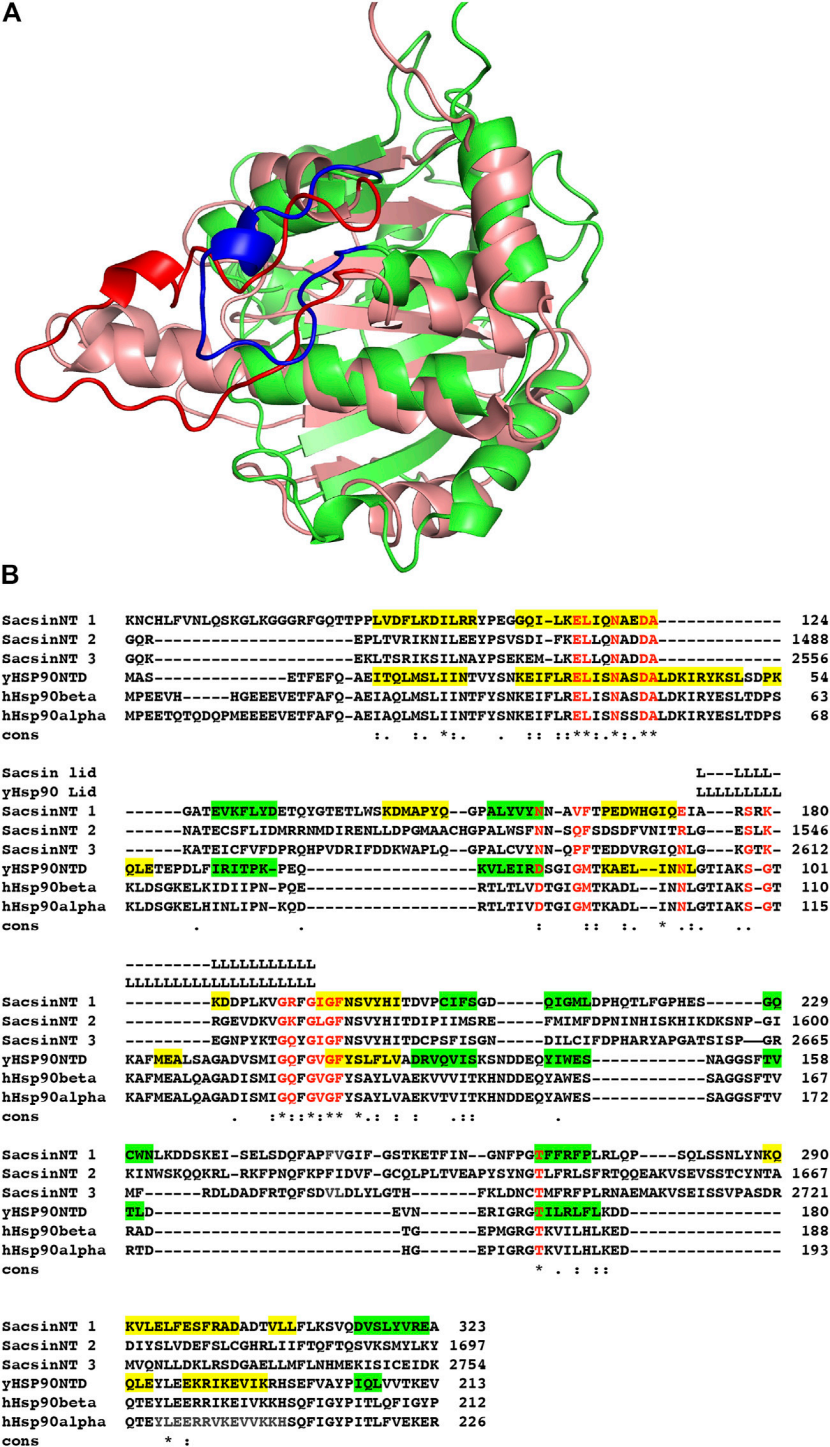
Superimposition of the sacsin SR1 and Hsp90 N-terminal domains. **(A)** The N-terminal domain of Hsp90 (salmon with red lid, PDB 2CG9) was superimposed onto the SR1 domain of the AlphaFold UniProt entry A0A804HIU0 (green with blue lid). **(B)** Sequence alignment of the N-terminal domains of yeast (yHsp90NTD) and human Hsp90 (hHsp90 α and β) with the three SR-domains of sacsin (SacsinNT 1–3). Green highlights, β-strand and yellow, α-helix. Cons, conservation, (.), weakly conserved residue position; (:) strongly conserved residue position and (*), invariant residue position.

Recently the structure of a region of human Sacsin (amino acids 1–177) has been determined using AlphaFold (Jumper et al., 2021; Varadi et al., 2022). Using the deposited structure, Uniprot A0A804HIU0, we have investigated whether the SR1 domains of the SIRPT regions of sacsin would be structurally consistent with the ATPase constraints of the Begarat fold. The crystal structure of the SR1 domain of sacsin (PDB 5V44) is essentially the same as that for the AlphaFold prediction, except that the crystal structure lacks details for the lid. In another deposited structure, PDB 5V46, the lid is almost intact, but not in a closed conformation and thus does not superimpose with the AlphaFold predicted structure. Using PyMOL (Schrödinger and Delano, 2020) to superimpose the N-terminal domain of AMPPNP bound Hsp90 (PDB 2CG9) with the SIRPT1-SR1 domain of sacsin, we were able to confirm that sacsin contains a Begarat fold, as reported earlier (Figure 2A) (Menade et al., 2018). Once superimposed, the peptide sequences between Hsp90 and sacsin were aligned based on a structural comparison and residues conserved in Hsp90 for ATP binding and hydrolysis were compared to residues in the sacsin sequence. We also confirm that the lid segment of the AlphaFold sacsin structure appears to be in a closed position, similar to that of Hsp90, but is apparently shorter in overall length, which influences the exact sequence conservation of ATP binding residues. However, the AlphaFold model confidence for the lid region is low, although this does not detract from the fact that it is a shorter segment of structure.

### Structure based alignment identifies residues required for ATP binding in the SR1 domain of sacsin

**FIGURE 3:**
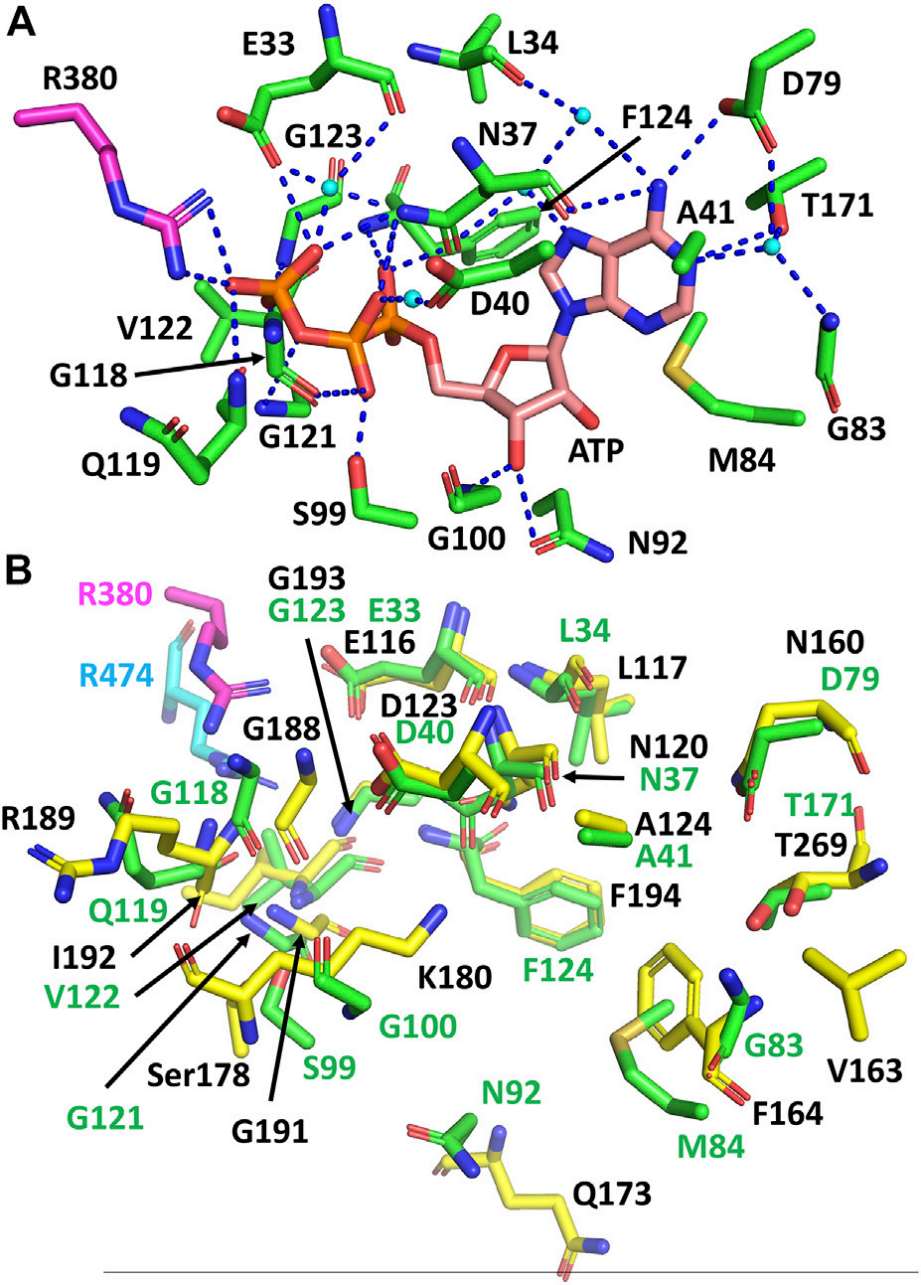
Comparison of the residues required for ATP binding and hydrolysis by Hsp90 and sacsin. **(A)** PyMOL cartoon of the yeast Hsp90 residues involved in the binding and hydrolysis of ATP. Residues are in stick conformation. The putative catalytic Arg 380 of Hsp90 is coloured in magenta. Water molecules are shown as cyan coloured spheres and hydrogen bond interactions as dashed blue lines. Potentially, these may also be conserved in sacsin, since the side chain environment around them is similar. **(B)** PyMol cartoon showing the superimposition of Hsp90 residues involved in the binding and hydrolysis of ATP and the corresponding sacsin residues. Green stick residues with green numbering, Hsp90 residues with the catalytic Arg 380 in magenta. Yellow stick residues with black numbering, sacsin residues with the catalytic Arg 474 shown in cyan.

Using the structurally aligned superimposition of Hsp90 and sacsin, we next compared the amino acid residues of Hsp90 involved in binding and hydrolysis of ATP with the peptide sequence of sacsin (Figure 2B). Using this alignment allowed us to accurately determine which sacsin residues correspond to ATP binding residues of Hsp90. The structurally based alignment shows that many of the residues required for ATP binding and hydrolysis are conserved in sacsin (Figure 3). The catalytic Glu 33 (sacsin Glu 116) as well as many other residues involved in binding of ATP are invariant (Table 1). These include the Hsp90 residue positions Leu 34, Asn 37, Asp 40, Ala 41, Gly 118, Gly 121, Gly 123, Phe 124 and Thr 171. Conserved residue positions include Asp 79, Met 84, Asn 92 and Ser 99 (see Table 1 for position of sacsin residues). Previously, it was claimed that Sacsin Phe 164 aligned with Hsp90 Gly 83 (Menade et al., 2018). However, our analysis suggests that Phe 164 replaces Hsp90 Met 84, allowing Phe 164 to perhaps pi-stack with the adenine ring of ATP. Consequently, Hsp90 Gly 83 aligns with SIRPT1-SR1 Val 163, where Val 163 could mimic the main-chain interactions formed by Gly 83, *via* a water molecule to Asp 79 and to a nitrogen atom of the adenine ring of ATP. Thus, the side chain of valine, would point away from the bound ATP and would not therefore interfere with its binding.

**TABLE 1:** A comparison of amino acid residue between yeast Hsp90 and the SIRPT1 to 3-SR1 domains of sacsin that are involved in ATP binding. Residues between Hp90 and the SIRPT1to 3-SR1 (HSP-NRDs) domains are either invariant or conserved and we comment on the feasibility of interaction at conserved positions.

Hsp90 N-terminal domain residues	SIRPT1-SR1 domain residue	SIRPT2-SR1 domain residue	SIRPT3-SR1 domain residue	Comment
Glu 33	Glu 116	Glu 1480	Glu 2548	Invariant
Leu 34	Leu 117	Leu 1481	Leu 2549	Invariant
Asn 37	Asn 120	Asn 1484	Asn 2552	Invariant
Asp 40	Asp 123	Asp 1487	Asp 2555	Invariant
Ala 41	Ala 124	Ala 1488	Ala 2556	Invariant
Gly 118	Gly 188	Gly 1554	Gly 2620	Invariant
Gly 121	Gly 191	Gly 1557	Gly 2623	Invariant
Glly 123	Gly 193	Gly 1559	Gly 2625	Invariant
Phe 124	Phe 194	Phe 1560	Phe 2626	Invariant
The 171	Thr 269	Thr 2642	Thr 2696	Invariant
Asp 79	Asn 160	Asn 1526	Asn 2592	conserved
Gly 83	Val 163	Gln 1529	Pro 2595	Val 163, Gln 1529 and Pro 2595 could all mimic main-chain interactions of Gly 83
Met 84	Phe 164	Phe 1530	Phe 2596	Phe 164 is perhaps involved in pi-pi stacking with the adenine ring of ATP. The MD simulation confirms this type of interaction.
Asn 92	Glu 174	Arg 1540	Asn 2606	Superimposition of secondary structural elements is poor in this region between Hsp90 and sacsin. MD simulations predict no interaction with this residue, but instead Asp 168 (Glu 88 in yeast Hsp90) fulfills this role instead.
Ser 99	Ser 178	Ser 1544	Gly 2610	Lid region AlphaFold model confidence is low
Gly100	Lys 180	Lys 1546	Lys 2612	Lid region AlphaFold model confidence is low. MD simulation predicts an inter-action of the lysine side chain with one of the g-phosphate oxygen atoms of ATP
Gln 119	Arg 189	Lys 1555	Gln 2621	Main chain interaction
Val 122	Ile 192	Leu 1558	Ile 2624	Main chain interaction

Other main chain contacts between Hsp90 and ATP include Val 122 that would allow substitutions in sacsin and Gly 100 which is replaced by Lys 180 in sacsin. Finally, the main chain of Hsp90 Gln 119 contacts ATP and this interaction could be maintained with the substitutions seen in sacsin (for example, Arg 189 in the SIRPT-SR1 repeat). Table 1 shows the residues positions for all three SIRPT-SR1 domains of sacsin that are equivalent to those found in Hsp90.

### The lid structure of the ATP binding pocket is shorter in sacsin than Hsp90

On closer inspection, the greatest variability in ATP binding residues between Hsp90 and sacsin are those that occur on the lid region of the SR domains. We noted that the AlphaFold model confidence for the lid structure of sacsin was low. Nonetheless the sacsin lid structure is significantly shorter than that of Hsp90 (Figure 2A). Sacsin residues that do not superimpose well with corresponding ATP binding residues of Hsp90 in the structure alignments include Ser 178 (Hsp90 Ser 99), Lys 180 (Hsp90 Gly 100), Gly 188 (Hsp90 Gly 118) and Arg 189 (Hsp90 Gln 119) (Figure 3B). The substitution of Gln 119 with sacsin Arg 189 can maintain the main chain interaction with ATP and the side chains of these amino acids are pointing away from the bound ATP. However, in the case of the substitution of Gly 100 with sacsin Lys 180, this at first sight appears to cause a clash with bound ATP. However, this residue position is solvent exposed, which might allow the side chain of Lys 180 to adopt a conformation that allows unhindered ATP binding, while maintaining a main chain contact with ATP. Alternatively, the side chain of Lys 180 might adopt one of a number of other possible conformations that could perhaps allow it to interact with sacsin Asn 120 and Asp 123 or even with the phosphate or 2′ and or 3′ hydroxyls of the ribose sugar of the bound ATP. However, the fact that in Hsp90 this position is an invariant glycine (Gly 100), does mean that a crystal structure or other further analyses is required to establish the exact consequences of this lysine substitution on the ATPase activity of these sacsin domains. Nonetheless, the misalignment of specific lid residues between Hsp90 and sacsin is consistent with the low confidence score for this region of the AlphaFold predicted structure. This means that it is likely that the model in this region of the AlphaFold structure either needs further refining or that the local structure of the lid is restructured upon ATP binding.

### Molecular dynamic simulations suggest a compatible ATP binding conformation for the lid region of sacsin

**FIGURE 4:**
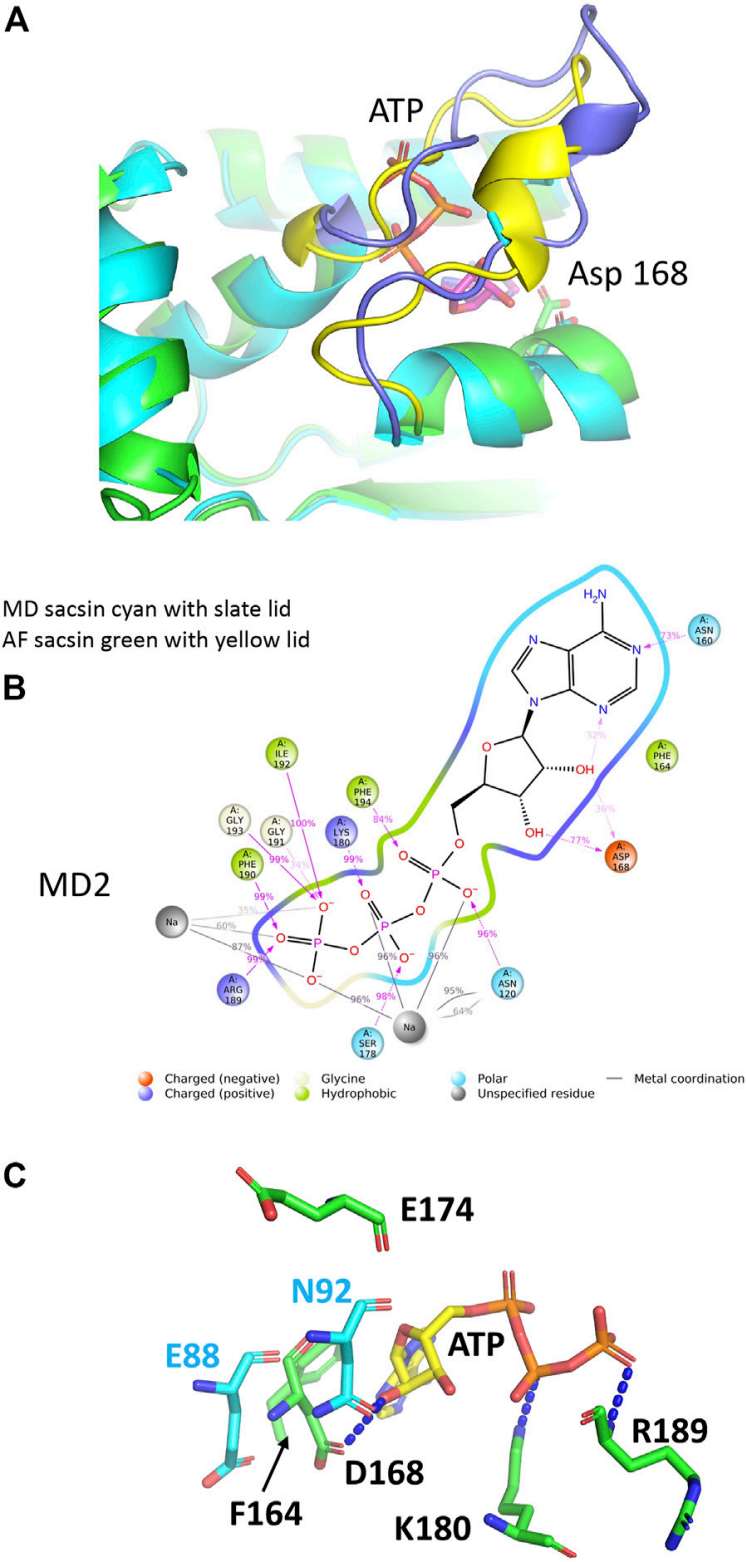
Analyses of the molecular dynamic model of sacsin-ATP. **(A)** Superimposition of the MD model of sacsin-ATP (cyan with slate coloured lid) with that of AlphaFold (green with yellow coloured lid). **(B)** A schematic of detailed ligand atom interactions with the protein residues. Interactions that occur more than 15.0% of the simulation time in the entire trajectory, are shown. It is possible to have interactions with >100% as some residues may have multiple interactions of a single type with the same ligand atom. Pi-cation interactions are shown in red, while hydrogen bond is shown in pink. **(C)** PyMol cartoon showing sacsin residues (green sticks) not conserved with Hsp90 and their interaction with ATP (yellow stick). Hsp90 residues are shown in cyan stick. Sacsin Asp 168 interacts with ATP, but the equivalent position in Hsp90, Glu 88 does not. In contrast, sacsin Glu 174 does not interact with ATP, but the equivalent Hsp90 residue, Asn 92, does interact.

Using molecular dynamics (MD) simulation (see Supplementary Material for methods) we set out to improve the structural model of the SR1 domain of sacsin bound with ATP. We investigated the lid’s dynamics through clustering analysis of the entire MD trajectory. We obtained five clusters summarizing the lid’s dynamics for the ATP-bound state, which populate one major conformation closed over the ligand (Supplementary Figure S1A). These results are reflected in the RMSD values of the protein, which is stable between 1.2 Å and 1.4 Å (Supplementary Figure S1B). The MD simulation for sacsin-ATP complex shows that ATP can establish a variety of interactions with numerous residues during the simulation (Supplementary Figure S1C). We observed that the lid is restructured (Figure 4A) and makes extensive interactions with ATP (Figure 4B). We noted that the main type of interactions are hydrogen bonds, followed by Coulomb interactions. The helix between residues Pro 166 to Gln 173 is also repositioned, which moves Asp 168 further back form the bound ATP, making it less likely to interfere with ATP binding.

On closer inspection we find that ATP particularly interacts with sacsin residues such as Asn 120, Asn 160, Phe 164, Asp 168, Ser 178, Lys 180, Arg 189, Phe 190, Gly 191, Ile 192, Gly 193 and Phe 194 through both hydrogen bonds, ionic interactions, and hydrophobic interactions. Details of single established interactions are reported in (Supplementary Figure S1C). Asn 120, Ser178, Phe 190, Ile 192, Gly 193 and Phe 194 represent residues conserved between sacsin and Hsp90 and are consistent in the specific type of interaction seen with ATP. Furthermore, sacsin Asn 160 replaces Hsp90 Asp79 and so maintains the critical interaction between this residue position and the exocyclic nitrogen atom of the adenine ring of ATP.

In the AlphaFold analysis we previously predicted that sacsin Phe 164 aligned with Hsp90 Met 84, allowing Phe 164 to perhaps pi-stack with the adenine ring of ATP. The MD simulation shows indeed that this is the case (Figure 4C). Another question that arose from the AlphaFold analysis was whether sacsin Arg 189 could form a contact with one of the γ-phosphate oxygen atoms of ATP as seen for Hsp90 Gln 119. The MD simulation also confirms that this substitution maintains a similar interaction with ATP (Figure 4C). Finally, the most controversial substitution was for sacsin Lys 180 for Hsp90 Gly 100. Our MD simulation suggest that the side chain of Lys 100 is involved in a side chain interaction with one of the γ-phosphate oxygen atoms of ATP (Figure 4C). Consequently, our MD simulation supports our interpretation of the interaction between ATP and sacsin and the slight structural arrangements in the lid segment of sacsin that we suggested form the AlphaFold analysis was corroborated by the MD simulation.

Finally, in Hsp90 Asn 92 forms a direct interaction with the 3′ hydroxyl group of the pentose ring of ATP (Figure 4C). However, the equivalent residue in sacsin, Asp 174, is unable to make such an interaction. Instead, the interaction comes from sacsin Asp 168, where the equivalent Hsp90 residue Glu 88, does not form the interaction. Thus, the interaction with the 3’ hydroxyl of the pentose ring of ATP is maintained by using two non-equivalent residue positions, namely sacsin 168 and Hsp90 Asn 92. On closer inspection the reason for this is that the helix (residues Pro 166 to Gln 173) that carries sacsin Asp 168 is slightly closer towards the bound ATP, than the equivalent helix in Hsp90 (residues Lys 86 to Leu 93) (Figures 4A,C). However, collectively our analysis indicates that sasin contains three Hsp-like N-terminal domains that are able to bind ATP.

#### The conserved residues, Arg 474, Arg 1839 and Arg 2893, from the SIRPT 1, 2 and 3 regions of sacsin share a common mechanistic role with the catalytic Arg 380 residue of Hsp90

**FIGURE 5:**
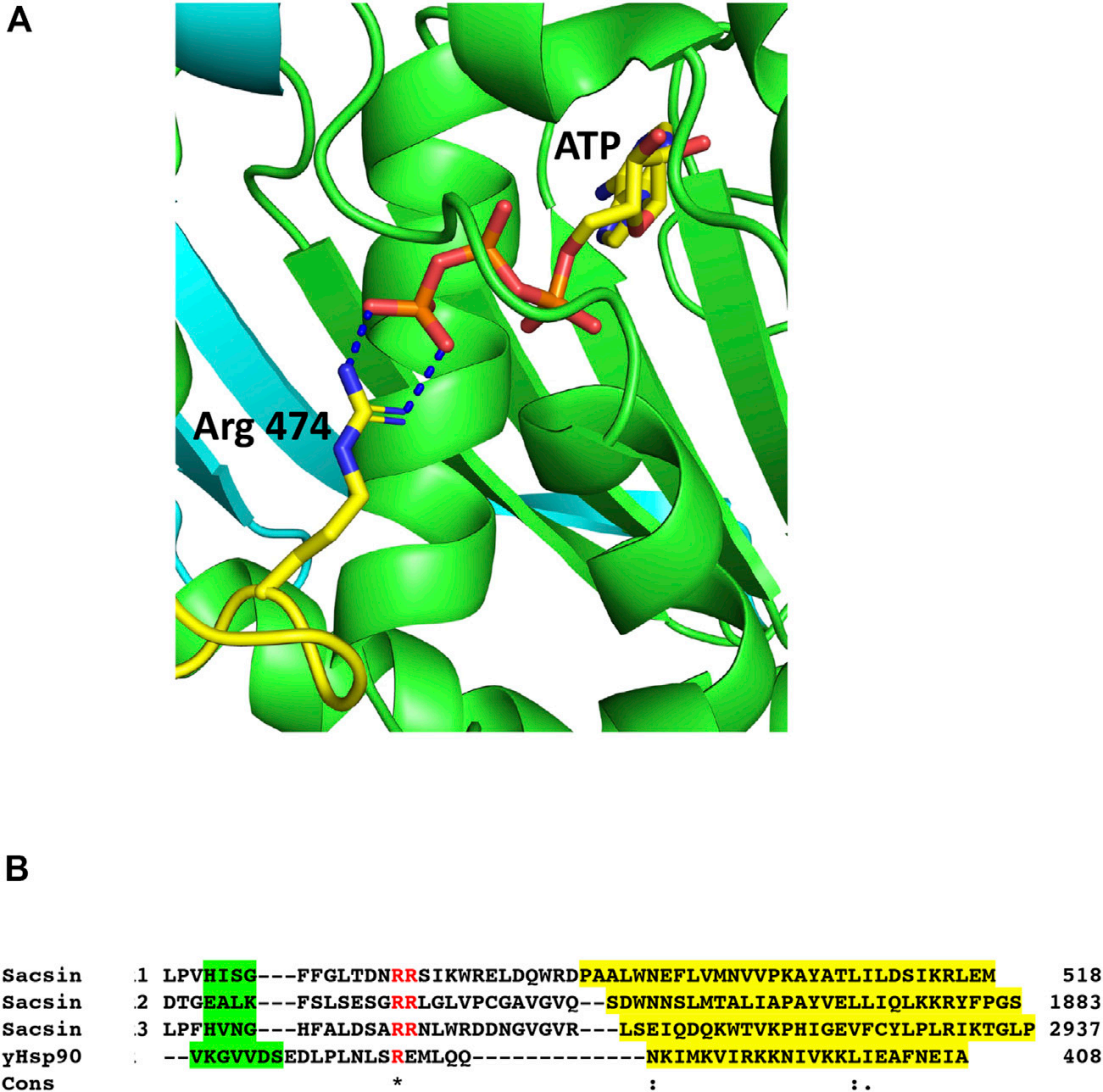
The catalytic Arg 474 of sacsin. **(A)** PyMol cartoon of the potential interaction of Arg 474 (yellow-blue stick molecule) with bound ATP (yellow-red stick molecule) in the SR1 domain (green). **(B)** Alignment of the catalytic loop regions, containing the conserved arginine residues of SR1, 2 and 3 (Sacsin 1, 2 and 3), and the adjoining structural elements with the same regions as for yeast Hsp90 (yHsp90). The conserved arginine residue and an adjacent arginine residue are shown in red. Structural elements are colour highlighted: Green, β-strand and yellow, α-helix. Cons, conservation, (.), weakly conserved residue position; (:), strongly conserved residue position and (*), invariant residue position.

With the N-terminal domain of Hsp90 correctly superimposed onto sacsin SIRPT-SR1, we were able to locate the putative catalytic arginine required for ATPase activity in the downstream (SIRPT1) region of sacsin, as observed in the Hsp90’s middle domain (Meyer et al., 2004). We were able to identify Arg 474 of sacsin as being orientated close enough to the superimposed Hsp90-bound ATP molecule such that it is potentially able to form similar contacts to ATP as seen with the putative catalytic Arg 380 of yeast Hsp90 (Figure 5A). We therefore propose that Arg 474, Arg 1839 and Arg 2893 (Table 1), which are conserved residue positions in the SIRPT1, 2 and 3 regions of sacsin respectively, are equivalent in function to Arg 380 of Hsp90 and are required by the SR1 domains of sacsin for efficient ATP hydrolysis (Figure 5B). Arg 380 is considered important as a putative catalytic residue because it contacts the γ-phosphate of ATP, but it is also known to directly interact with the catalytic glutamate of Hsp90 (Ali et al., 2006). It is therefore likely to influence catalysis to some degree, although it has been argued that it plays a greater role in stabilising the N- and middle-domain interactions required to form a catalytically active state (Ali et al., 2006; Cunningham et al., 2012), and thus distinguishing its precise role is complex. However, the alignment of the putative arginine catalytic loop, and the structural elements on either side of this loop, are presented in Figure 5B, which shows little peptide sequence conservation with yeast Hsp90. The conservation of Arg 474, 1839 and 2893, suggests that sacsin would be an active ATPase. Furthermore, the R474C mutation in sacsin has been reported to score as highly as truncated forms of sacsin on the SPAX Scoring System, suggesting that R474C mutant is non-functional [19]. This provides further support that sacsin is an active ATPase protein.

#### Structural similarity exists between the middle domain of Hsp90 and sacsin SIPRT domains

**FIGURE 6:**
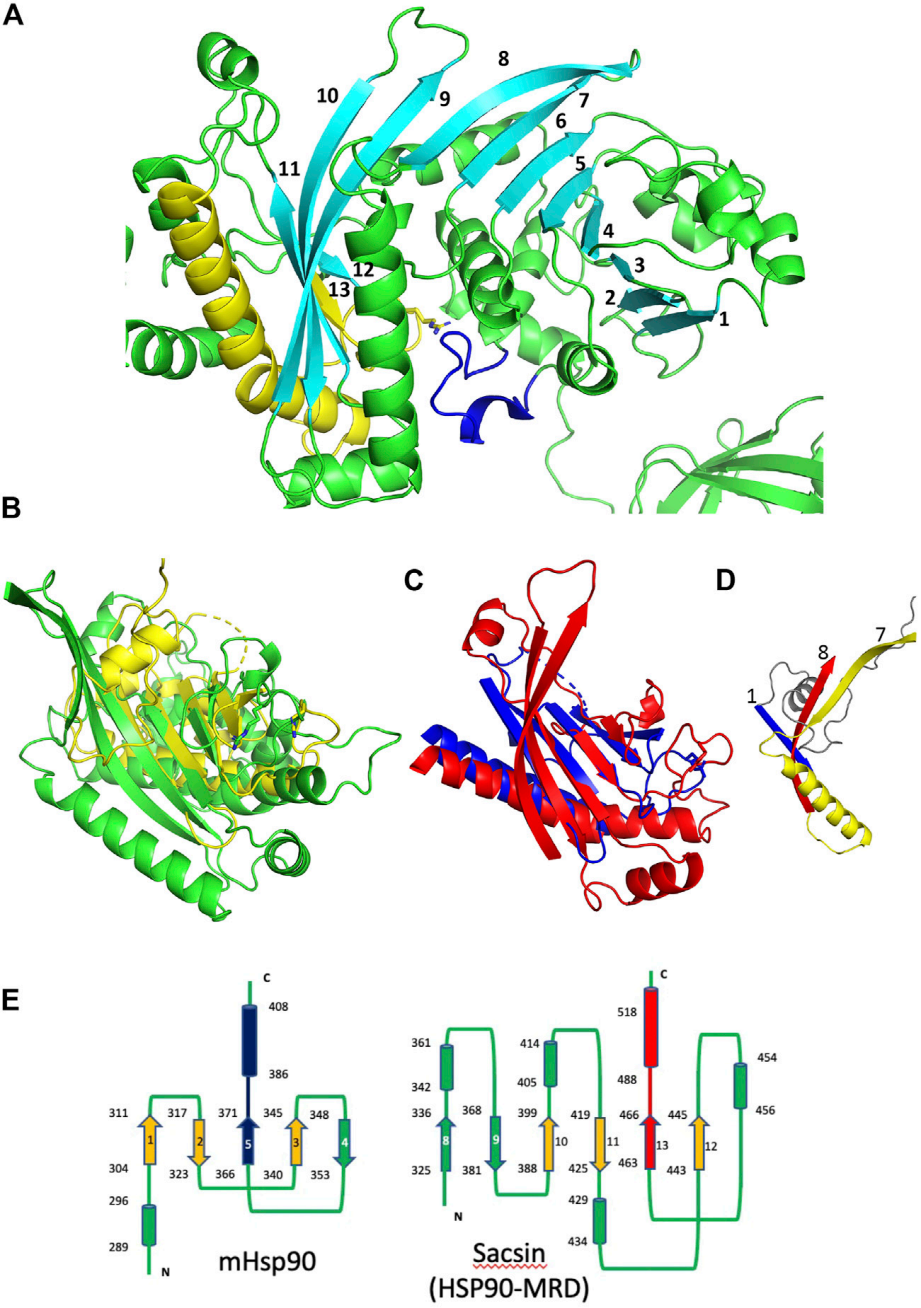
Comparison of the Hsp90 middle domain with corresponding sacsin regions. **(A)** PyMol cartoon of the SR1 domain and immediate downstream region of sacin (green and cyan). A continuous 13 stranded β-sheet runs from the SR1 domain and into the adjacent domain of sacsin. Cyan, β-strands, yellow, the β-strand and helix flanking the catalytic loop (yellow). Blue, the lid segment of the SR1 domain of sacsin. **(B)** PyMol cartoon showing the superimposition of the Hsp90 middle domain (yellow, residues 262-444) and the corresponding sacsin region (green, residues 325-518). **(C)** The central core structural elements of the middle domain of Hsp90 (blue) and the corresponding region of sacsin (red) show the main elements that superimpose. **(D)** PyMol cartoon showing the N-terminal structural elements of the Hsp90 middle domain (blue and grey) and the corresponding elements of sacsin (red and yellow), showing that these structural elements do not superimpose. Strand 7 and 8, sacsin SR1 β-strands and strand 1, Hsp90 middle domain β-strand. **(E)** Topology diagrams for the middle domain of Hsp90 (left panel) and the corresponding region of sacsin (right panel, HSP-Middle Repeat Domain (HSP-MRD)). Cylinders, α-helix, arrows, β-strand and lines are connections between the structural elements. The start and end residue numbers of each structural element are shown, as are the β-strand numbers. Blue and red, structural elements that represent the arginine catalytic loop and the flanking structural elements. Alignment of the red and blue β-strand and α-helix allows the superimposition of the orange β-strands.

In order to obtain the best superimposition of the middle domain of Hsp90 with the equivalent region of sacsin, an orientation of the Hsp90 middle domain (residues 262 to 444 used) was required. The middle domain of Hsp90 contains a 7-stranded β-sheet, whereas sacsin contains a continuous 13-stranded β-sheet that runs from the SR1 domain and into the downstream SIRPT1 region, of which the last 5 β-strands appear to form the central core of the domain (Figure 6A). To get the best superimposition of the middle domain of Hsp90 (residues 262–408) with sacsin (residues 325–518), we orientated the Hsp90 middle domain β-sheet such that we could match it with that of sacsin (Figure 6B). The best alignment maintained the antiparallel and parallel nature (single pair of strands) of the β-strands that formed each β-sheet and allowed the longest helix of both the Hsp90 middle domain and the equivalent sacsin segment to be approximately aligned (Figure 6C). From this superimposition, a central core of structure that could be considered similar was identified (Figures 6C,D), which was otherwise impossible by standard automated superimposition techniques. Collectively this included the superimposed β-strands and the long helix of these domains. Most importantly, the structural elements on either side of the putative arginine catalytic loop, which consists of one of the superimposed β-strands and the following superimposed long helix of these domains were matched well. This suggested a similar sub-structure (Figure 6C) in what otherwise appears to be an unrelated fold at first sight (Figure 6B). The topology of the Hsp90 middle domain and the corresponding sacsin region is shown in Figure 6E. Consequently, we identify sacsin residues 325 to 518 as representing a Hsp90-like middle domain, which corresponds to a fragment of the Hsp90 middle domain (residues 262–408). We propose that the SR1 regions of sacsin be renamed as the HSP-NRD (Hsp90 N-terminal Repeat Domain; residues 84-324) and the fragment immediately downstream as HSP-MRD (Hsp90 Middle Repeat Domain; residues 325-518). Residues immediately after position 518 of sacsin do form α-helices, as seen in the Hsp90 middle domain, but our alignments of these secondary structural elements did not superimpose well.

Further analysis of the C-terminal domain of Hsp90 with topologically corresponding regions of sacsin, did not appear to show any structural homology (Supplementary Figure S2). We therefore conclude that the SR1 domains of sacsin are homologues to the N-terminal domains of Hsp90, that the Hsp90 middle domain (residues 262–408) is structurally similar to a central core of the corresponding sacsin region, but the remaining section of Hsp90, including the C-terminal domain appears to be very different. Nonetheless, it is clear that sacsin possesses a similar catalytic loop sub-structure that provides and orientates the putative catalytic arginine for catalysis.

#### The AlphaFold model predicts that Hsp90 inhibitors may not bind sacsin because of steric hinderance from Asp 168

**FIGURE 7:**
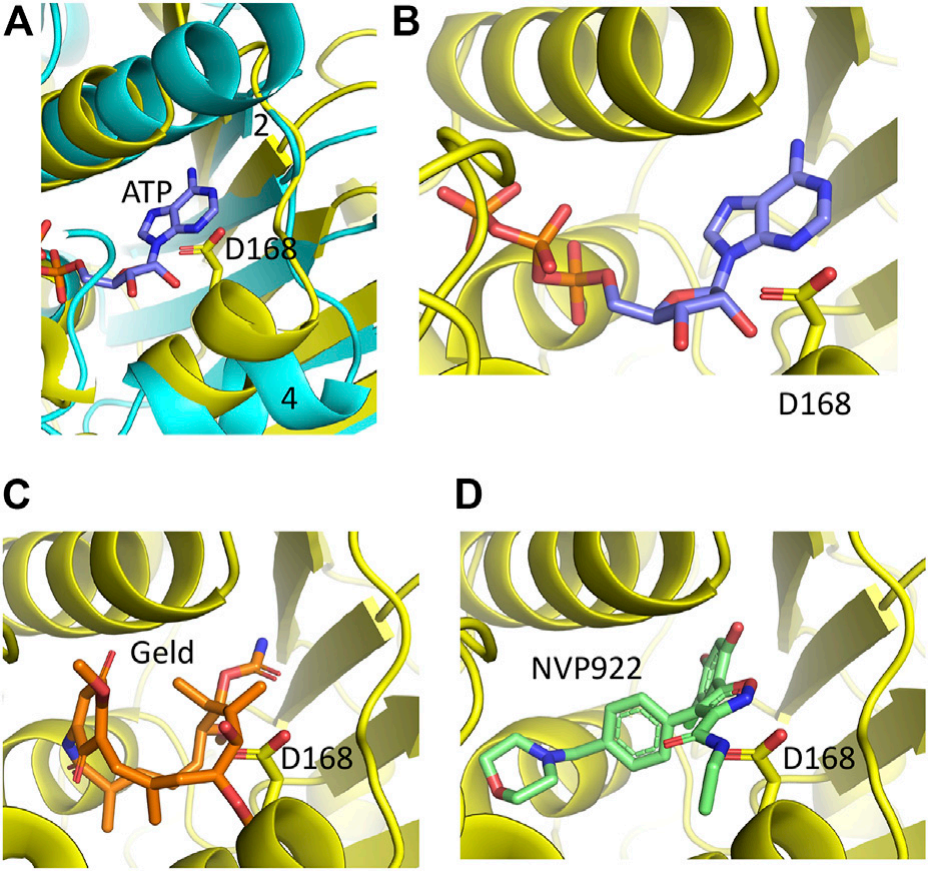
PyMol cartoons of Hsp90 N-terminal domain inhibitors superimposed into sacsins ATP binding pocket. **(A)** α-Helix 4 of Hsp90 (cyan) is positioned to create a more open ATP-binding site. In contrast, the equivalent α-helix in sacsin (yellow) restricts the opening of the ATP-binding pocket. Strand 2, β-strand 2 of Hsp90 is indicated. **(B)** ATP docked into the ATP-binding site of sacsin, showing that it avoids any obvious clashes with the side chain of Asp 168. **(C)** Geldanamycin docked into the ATP-binding site of sacsin, showing that it clashes with the side chain of Asp 168. **(D)** AUY922 docked into the ATP-binding site of sacsin, showing that it clashes with the side chain of Asp 168.

Hsp90 can be targeted by drugs that inhibit its ATPase activity through competitive binding of the nucleotide binding pocket (e.g., radicicol, geldanamycin and its analog tanespimycin/17-AAG) (Armstrong et al., 2018). Given the homology between the Hsp90 ATP binding domain and the sacsin SR1 domains there is a possibility these inhibitors, or related drugs, may target sacsin. This has previously been investigated in an *in vitro* ATPase assay which saw no effect of geldanamycin or radicicol on activity of a region of mouse sacsin from the N-terminus of the protein to the beginning of the second SIRPT domain (Anderson et al., 2010). To provide a structural explanation of why these inhibitors appear not to affect sacsin we modelled superimposition of the N-terminal domain of Hsp90 containing geldanamycin and AUY922 into sacsin’s ATP binding pocket (Figure 7). In Hsp90, the loop connecting β-strand 2 and the following α-helix consists of a total of 8 residues (positions 79–86). In contrast, sacsin has a shorter loop, consisting of just 6 residues (positions 160–166). The consequence of this is that the α-helix connected to this loop is drawn closer to bound ATP. While its effect on ATP binding appears negligible, it could result in clashes with geldanamycin and AUY922 and in particular with the side chain of sacsin Asp 168 (Figure 7). This suggests that these inhibitors may not be able to bind directly to the ATPase site of sacsin.

#### Molecular dynamic simulations suggest that some Hsp90 inhibitors may be able to bind sacsin

MD simulations for the sacsin-geldanamycin complex were carried out as described for the ATP bound structure of the Hsp-NRD of sacsin (see Supplementary Material).

In order to investigate the motions of the loop in our simulations, we carried out clustering analysis on the entire trajectory. We observed that the loop explores virtually a single conformation, closed over the ligand, during the simulation time (Supplementary Figure S3A). Otherwise, geldanamaycin induces some small rearrangements which raise RMSD up to 2 Å (Supplementary Figure S2B). The protein maintains its overall stability.

**FIGURE 8:**
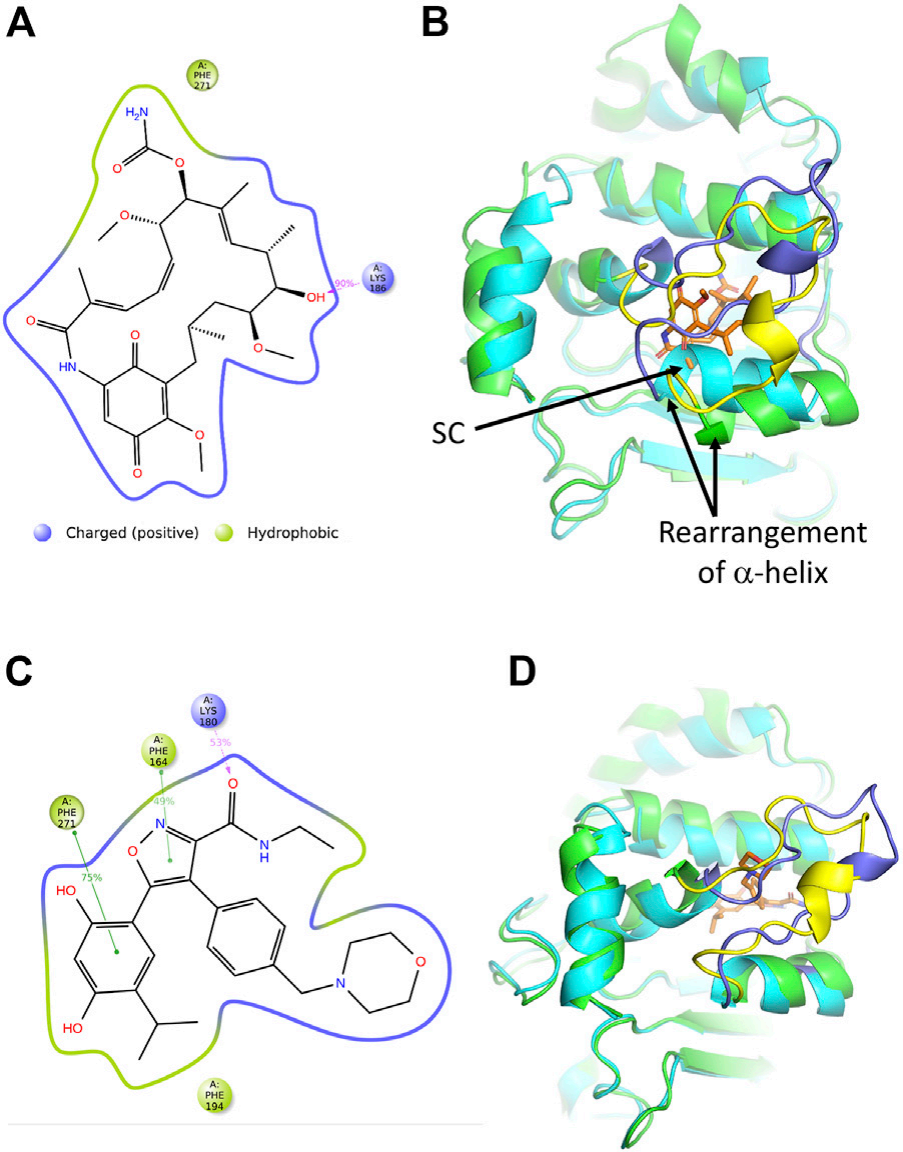
Analyses of the molecular dynamic model of sacsin-geldanamycin and sacsin-AUY922. **(A)** A schematic of detailed ligand atom interactions with the protein residues. Interactions that occur more than 20.0% of the simulation time in the entire trajectory, are shown. It is possible to have interactions with >100% as some residues may have multiple interactions of a single type with the same ligand atom. Hydrogen bond is shown in pink. **(B)** Superimposition of the MD models of sacsin-ATP (cyan with slate coloured lid) and sacsin-geldanamycin (green with yellow coloured lid), showing how a helix of sacsin is rearranged in order to accommodate geldanamycin. **(C)** A schematic of detailed ligand atom interactions with the protein residues. Interactions that occur more than 30.0% of the simulation time in the entire trajectory, are shown. It is possible to have interactions with >100% as some residues may have multiple interactions of a single type with the same ligand atom. Hydrophobic interactions (such as pi-pi stacking) are shown in green, while hydrogen bond is shown in pink. **(D)** Superimposition of the MD models of sacsin-ATP (cyan with slate coloured lid) and sacsin-AUY922 (green with yellow coloured lid). B, Superimposition of the MD models of sacsin-ATP (cyan with slate coloured lid) and sacsin-AUY922 (green with yellow coloured lid).

Results from the protein-ligand interactions analysis highlight that the most recurring interaction is a hydrogen bond between the ligand’s hydroxyl and the lid residue Lys 186, which is conserved during the majority of the simulation (90% of simulation time) (Supplementary Figure S3C and Figure 8A). Another non-lid interaction that can be observed from the graph is an unspecific hydrophobic interaction involving residue Phe 271. An additional feature of the MD simulation with geldanamycin is that it requires some structural rearrangements to accommodate its binding. Binding to the structure form the sacsin-ATP MD simulation indicates a steric clash (Figure 8B). Whether geldanamycin can therefore freely bind the HSP-NRD of sacsin remains questionable.

MD simulations for the sacsin-AUY922 complex were also carried out as described for the ATP bound structure of the HSP-NRD of sacsin (Supplementary Material). In order to investigate the motions of the lid in our simulations, we carried out clustering analysis on the entire trajectory, consistent with the analysis of the ATP-bound case (Supplementary Material). As with geldanamycin, we observed that the loop explores virtually a single conformation, closed over the ligand, during the simulation time (Supplementary Figure S4A). This observation is in accordance with the overall low value of protein RMSD (Supplementary Figure S4B).

Protein-Ligand interactions analysis identify four key interactions established by AUY922 in the binding site. These interactions are categorized by type and summarized in Supplementary Figure S4C. In detail, residue Phe194 establishes an unspecific hydrophobic interaction with the AUY922 isopropyl moiety (Figure 8C). Otherwise, residues Phe 164 and Phe 271 are involved in pi-pi stacking interactions with the ligand’s isoxazole ring (49% of simulation time) and catechol moiety (75% of simulation time), respectively (Figure 8C). Moreover, the AUY922 amide establishes a hydrogen bond with Lys 180 (53% of simulation time), which belongs to the investigated loop (Figure 8C). However, in contrast to geldanamycin, AUY922 appears to require minimal structural rearrangements relative to the sacsin-ATP complex (Figure 8D).

In conclusion, it appears that sacsin is able to bind and hydrolyse ATP because a full complement of the machinery required for binding and catalysis is present. We see a few non-conserved positions, but the models explain how these residue differences with Hsp90 can still interact with ATP. In contrast, we are less certain if the Hsp90 ATPase inhibitors, geldanamycin and AUY922, are able to freely bind the Hsp-NRD’s of sacin.

### Potential for sacsin HSP-NRD dimerization

**FIGURE 9:**
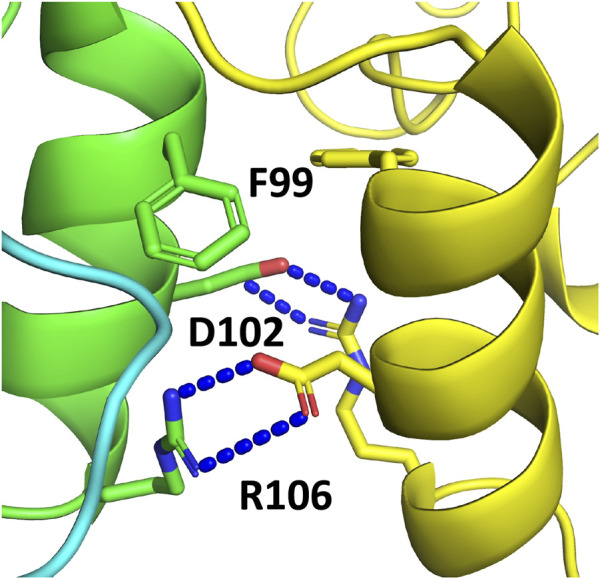
AlphaFold prediction of the dimerization interface of the SIRPT1 HSP-NRD domain of sacsin. The interface is clearly modelled on the dimerization of the N-terminal domains of Hsp90. Resides important in the dimerization of the HSP-NRDs are shown in stick format.

In order to investigate whether sacsins HSP-NRD could dimerise, as seen with the N-terminal domains of Hsp90, we used AlphaFold to generate models using Colab’s accelerated prediction from within ChimeraX (Goddard et al., 2018; Jumper et al., 2021; Pettersen et al., 2021; Mirdita et al., 2022; Varadi et al., 2022). We found that AlphaFold predicted a dimeric structure for the HSP-NRD domain similar to that of the N-terminal domains of Hsp90 (Figure 9). At the core of the interface, we found the symmetrically opposed hydrophobic side chain of Phe 99 and the side chains of Asp 102 and Arg 106 forming ionic interactions (Figure 9). While at first sight this may seem encouraging, the limitations of AlphaFold currently suggest that about one-third of interfaces are incorrect in multimer predictions. Thus, we critically analysed the residues forming the dimeric interface of the HSP-NRD. Sacsin Phe 99 is represented by an equivalent conserved Leucine residue in Hsp90 (Leu 15 in yeast) and would be compatible with dimerization. However, unlike the first HSP-NRD, the second and third HSP-NRDs contain an Arginine residue (Arg 1,643 and Arg 2540, respectively) instead of phenylalanine, which would be hard to reconcile within the dimer interface. Similarly, HSP-NRD Asp 102 residue is represented by an equivalent conserved Leucine in Hsp90 (Leu 18 in yeast) and is either Asn 1,646 or Serine 2,543, in the subsequent HSP-NRDs, respectively. The lack of conservation suggests that perhaps the HSP-NRD do not dimerise. Similarly, the HSP-NRD Arg 106 residue, which is represented by a equivalent conserved Threonine in Hsp90 (Thr 22 in yeast), is either Glu 1,650 or Ala 2547 in the second and last HSP-NRDs of sacsin, respectively. In fact, AlphaFold models of the second and third HSP-NRD, each represented as a dimer, show that the interface for these models does not appear to form any sort of coherent hydrophobic core and the arginine residues, Arg 1,643 (HSP-NRD2) and Arg 2,540 (HSP-NRD3), would not easily allow such an dimeric interface to form (results not shown). Thus, collectively these results suggest that while the first HSP-HRD of sacsin may be able to dimerize, the lack of conservation between all three HSP-NRDs suggests that this does not seem to be the case. Clearly, further biochemical or structural studies are required to determine if any sort of HSP-NRD dimerization is part of the chaperone cycle of these domains.

## Discussion

This analysis suggests that sacsin contains three Hsp90-like N-terminal- and middle-domains, which we designate the HSP-NRD and HSP-MRD segments. We hypothesise that together they are responsible for an ATPase activity. Our alignments suggest that the lid segment of sacsin is about 12 residues shorter than for Hsp90 and this consequently results in some alteration of residues at specific positions that are required to maintain ATP binding. This is very apparent for the invariant Gly 100 of Hsp90, which is replaced by Lys 180 in SIRPT1-SR1 of sacsin. ARSACS causing mutations are found in the predicted ATP binding regions of sacsin, including mutations that would be predicted to specifically inhibit ATPase activity (e.g., R474C). This supports the notion that sacsin requires ATP activity for its function.

The ATPase activity of Hsp90, and thus its function, is partly determined by dimerization mediated through the C-terminal domain (Prodromou et al., 2000; Ali et al., 2006; Wayne and Bolon, 2007). Crystallographic structural analysis of sacsin’s C-terminal HEPN domain (Kozlov et al., 2011) also identified a dimer, suggesting the full-length protein might be dimeric. If this is the case, it is possible that dimerization of sacsin may influence the ATPase activity of its SR1 domains and ultimately its function. However, the AlphaFold structure shows that the lid segment of sacsin’s HSP-NRD is packed against α-helix 1 and 2, as well as the loop following α-helix 2, of the HSP-MRD. Consequently, this may indicate that direct dimerization of HSP-NRDs is not required for ATPase activity. In fact, we find that residues that would be involved in a dimeric interface between HSP-NRDs (based on the Hsp90 N-terminal dimerization model) are not conserved in the three HSP-NRDs of sacsin. Instead, we find that α-helix 1 and 2 of the HSP-MRD, which represents a structural element that does not superimpose with the Hsp90 middle domain, may substitute for HSP-NRD dimerization, providing stability for the lid segment in the closed ATP state. In contrast, Hsp90’s lid is mostly stabilised by the N-terminal domain from the adjacent protomer of the Hsp90 dimer. Thus, it appears that the stabilization of sacsin’s lid segments may be very different to that for Hsp90. However, assuming that dimerization of the Hsp90-like segments of sacsin is not required for its function, the question arises whether the mechanism of action between these chaperones is similar. Hsp90 has been seen to unfold clients such as kinases, with both protomers of the Hsp90 dimer involved in separating the N- and C-lobes of the kinase domain (Verba et al., 2016). Alternatively, sacsin may use its individual Hsp90-like domains, that are spatially separated, to achieve a similar effect on its clients, as seen with the Hsp90-kinase complex. Clearly, our analysis of sacsin structure has raised multiple intriguing questions that will require both biochemical and structural determinations to define mechanism.

It is a common feature of chaperone machines that they are driven by ATP binding and hydrolysis to assist protein folding and unfolding (Clare and Saibil, 2013). Therefore, our analysis would be concordant with sacsin functioning as an ATP-driven molecular chaperone. If this is the case a key challenge will be to identify sacsin’s clients. One candidate group of proteins are intermediate filaments, which have been identified in a sacsin interacome (Romano et al., 2022). Moreover, significant reorganisation of the intermediate filament cytoskeleton is observed in sacsin null cells (Girard et al., 2012b; Lariviere et al., 2015; Bradshaw et al., 2016; Duncan et al., 2017; Gentil et al., 2019). This includes the formation of perinuclear accumulations of vimentin in sacsin knockout SH-SY5Y cells and ARSACS patient dermal fibroblasts (Duncan et al., 2017), as well as abnormal bundling of non-phosphorylated neurofilament in neurons from sacsin knockout mice (Lariviere et al., 2015) and aggregation of glial fibrillary acidic protein in glial cells (Murtinheira et al., 2022). Unexpectedly, heterologous expression of isolated sacsin domains can modulate neurofilament assembly (Lariviere et al., 2015). This includes the isolated UBL, SIRPT 1 and J-domains, which all modified neurofilament assembly *in vivo*, with the SIRPT1 and the J-domain were having opposing effects, by respectively promoting and preventing filament assembly. The intermediate filament phenotype of motor neurons from the sacsin knockout mice was also altered by expression of the sacsin SIRPT1 or J-domain, with partial resolution of existing neurofilament bundles. That these isolated domains of sacsin influence neurofilament organisation is perhaps surprising but would again be consistent with the full-length protein functioning as a chaperone for intermediate filament assembly or disassembly.

Hsp90 works with other chaperones and cochaperones as part of a larger protein folding and remodelling machinery. Of particular importance is Hsp90’s collaboration with Hsp70 in protein folding and other chaperone functions (Genest et al., 2019). The presence of the J-domain in sacsin implies that it also requires a Hsp70 partner for its function. It also seems likely that if sacsin is an ATP-driven chaperone then its function could be regulated by interacting partners acting as cochaperones, as is the case for Hsp90 and other chaperones. More evidence for sacsin functioning in a chaperone network comes from its putative interactome which includes a chaperone cluster (Romano et al., 2022). Our hypothesis therefore suggests that sacsin acts as a central hub of chaperone activity, which could define it as a super molecular chaperone complex. However, the modelling generated hypothesis presented here will ultimately require validation, which for a large protein such as sacsin will be challenging, although the Hsp90 modules we define here should help reduce the complexity of such biochemical and structural studies.

Finally, our analysis indicates that for the Hsp90 inhibitors we investigated, which target its ATP-binding site, AUY922 may bind sacsin, whereas geldanamycin may not. This is important as these drugs are in clinical trials as therapeutics for cancers and other diseases, such that off target effects on sacsin would be undesirable. However, further work is required to establish whether sacsin is a target of specific Hsp90 ATPase inhibitors.

## Data Availability

Publicly available datasets were analyzed in this study. This data can be found here: The link to the AlphaFold structure, https://www.uniprot.org/uniprotkb/A0A804HIU0/entry; PDB 5V44, https://www.rcsb.org/structure/5V44; PDB 5V46, https://www.rcsb.org/structure/5V46 and 2CG9, https://www.rcsb.org/structure/2CG9.

## Supplementary material

Supplementary Figure S1 Molecular Dynamic simulation of sacsin-ATP complex. **(A)** Cluster analysis of the loop; the most populated cluster is shown in red, the second in orange, the third in yellow, the fourth in green and the least populated in blue. **(B)** RMSD analysis of protein C-alpha (blue) and ligand (magenta). **(C)** Protein-ligand interaction diagram. Interactions are categorized into four types: Hydrogen Bonds (green), Hydrophobic (lilac), Ionic (pink) and Water Bridges (blue). The stacked bar charts are normalized over the course of the entire trajectory. Values over 1.0 are possible as some protein residue may make multiple contacts of same subtype with the ligand.

Supplementary Figure S2 Hsp90 middle and C-terminal regions and corresponding segments of sacsin that do not superimpose. PyMol cartoon of Hsp90 (yellow and blue, residues 386 to 677) and sacsin (green and red, residues 487 to 772). The blue long helix of the Hsp90 middle domain and that of sacsin are superimposed. The rest of the structure shows no immediately recognisable common topology.

Supplementary Figure S3 Molecular Dynamic simulation of sacsin-geldanamycin complex. **(A)** Cluster analysis of the loop; the most populated cluster is shown in red, the second in orange, the third in yellow, the fourth in green and the least populated in blue. **(B)** RMSD analysis of protein C-alpha (blue) and ligand (magenta). **(C)** Protein-ligand interaction diagram. Interactions are categorized into four types: Hydrogen Bonds (green), Hydrophobic (lilac), Ionic (pink) and Water Bridges (blue). The stacked bar charts are normalized over the course of the entire trajectory. Values over 1.0 are possible as some protein residue may make multiple contacts of same subtype with the ligand.

Supplementary Figure S4 Molecular Dynamic simulation of sacsin-AUY922 complex. **(A)** Cluster analysis of the loop; the most populated cluster is shown in red, the second in orange, the third in yellow, the fourth in green and the least in blue. **(B)** RMSD analysis of protein C-alpha (blue) and ligand (magenta). **(C)** Protein-ligand interaction diagram. Interactions are categorized into four types: Hydrogen Bonds (green), Hydrophobic (lilac), Ionic (pink) and Water Bridges (blue). The stacked bar charts are normalized over the course of the entire trajectory. Values over 1.0 are possible as some protein residue may make multiple contacts of same subtype with the ligand.
