# Supplementary material for: AlphaFold predicted structure of the Hsp90-like domains of the neurodegeneration linked protein sacsin reveals key residues for ATPase activity
Source: Front Mol Biosci. 2023 Jan 13;9:1074714. doi: 10.3389/fmolb.2022.1074714 (PMC9880540; doi:10.3389/fmolb.2022.1074714)
Supplement: Supplementary file 1 [file DataSheet1.pdf]

Supplementary Material

Methods for Molecular Dynamic Simulations

In order to help resolve the issue that Lys 180 is compatable with ATP binding we used molecular dynamic simulation to determine if the lid region of sacsin could be compatable with bound ATP. The starting structure was prepared using the Protein Preparation Wizard tool in Schrodinger-Maestro suit (1, 2). Original hydrogens were removed, and protonation states were optimized at pH = 7 +/– 2 (Glu, Asp, His tautomer) with PROPKA. Then, H-bond geometries were adjusted considering the calculated protonation states. Water molecules were removed except for the ones placed in a 6 Å shell centred on the ligand (ATP, AUY922, or Geldanamaycin). Ligands were prepared using the LigPrep tool in Schrodinger-Maestro suite (3), protonation states were calculated at pH = 7 +/– 2 with PROPKA.

The systems obtained through this procedure were solvated in a cubic box of 20 Å using TIP3P water molecules and neutralized with the required number of Na^+ cations using the “System Builder” tool in Desmond (4). Both the protein and ligand were parametrized using OPLS4 force field (5).

In order to optimize the active site structure, we minimized the system (100 ps) with a soft restraint (5 kcal/mol) on the ligand and on residues not belonging to the binding site (defined by a shell of 6 Å from the ligand). In other words, residues within 6 Å from the ligand were free to move, whereas the rest of the protein and the ligand were restrained. In this framework, minimization uses a hybrid method of the steepest decent and the limited-memory Broyden-Fletcher-Goldfarb-Shanno (LBFGS) algorithms. Since both ATP and Geldanamycin present many steric clashes in the starting systems, three additional steps were carried out: 1), 100 ps of minimization with soft restraint (5 kcal/mol, using a hybrid method of the steepest decent and the LBFGS algorithms) on the whole protein, leaving the ligand free to move. 2), 1 ns of minimization of the whole system without restraints. 3), 10 ns of molecular dynamics simulation with soft restraint (0.5 kcal/mol) applied on the ligand.

After the minimization, Molecular Dynamic (MD) simulation was performed using Desmond (4): the recording interval was set to 50 ps and the overall simulation time was 500 ns, producing a simulation of 10000 frames. The NPT ensemble was chosen with T = 300 K and P = 1 atm. Before trajectory production, the following (standard) relaxation protocol was carried out: 1), 100 ps of Brownian dynamics in the NVT ensemble at 10 K with restraint on solute heavy atoms (force constant = 50.0 kcal/mol). 2), 12 ps simulation in the NVT ensemble at 10 K (Langevin thermostat, τ = 0.1; (6) with restraint on solute heavy atoms (force constant = 50.0 kcal/mol). 3), 12 ps simulation in the NPT ensemble at 10 K (Langevin thermostat, τ = 0.1;(6) and 1 atm (Langevin barostat, τ = 50.0; (7)) with restraint on solute heavy atoms (force constant = 50.0 kcal/mol). 4), 12 ps simulation in the NPT ensemble at 300 K (Langevin thermostat, τ = 0.1; (6)and 1 atm (Langevin barostat, τ = 50; (7)) with restraint on solute heavy atoms (force constant = 50.0 kcal/mol). 5), 24 ps simulation in the NPT ensemble at 300 K (Langevin thermostat, τ = 0.1; (6) and 1 atm (Langevin barostat, τ = 2.0; (7)).

Once the minimization, heating and equilibration steps are done, the MD production starts and lasts for 500 ns at 300 K (MTK thermostat, τ = 1.0) and 1 atm (MTK thermostat, τ = 1.0).

Protein-ligand interactions analysis were performed using the “Simulation interaction diagram” tool and PyMol analysis of the structures. Interactions are categorized into four types: Hydrogen Bonds (green), Hydrophobic (lilac), Ionic (pink) and Water Bridges (blue). These interactions are summarized in stacked bar charts and normalized over the course of the entire trajectory. Here, a value of 0.5 indicates that a specific interaction is maintained for 50% of the simulation time. Values over 1.0 are possible as some protein residue may make multiple contacts of same subtype with the ligand. Clustering analyses were carried out with “Desmond trajectory clustering” tool. RMSD-based matrix was centred on residues 176-191 was carried out on the whole trajectory (every frame was considered) and 5 clusters were identified.

References

1. Schrödinger: MacroModel. Schrödinger LLC. New York, NY (2021)

2. Schrödinger: Epik, Schrödinger, LLC; Impact, Schrödinger, LLC, New York, NY; Prime, Schrödinger, LLC, New York, NY.Protein Preparation Wizard (2021)

3. Schrödinger: LigPrep. Schrödinger LLC. New York, NY (2021)

4. D. E. S. Research: Desmond Molecular Dynamics System. New York, NY. Maestro-Desmond Interoperability Tools, Schrödinger, New York, NY. (2021)

5. C. Lu, C. Wu, D. Ghoreishi, W. Chen, L. Wang, W. Damm, G. A. Ross, M. K. Dahlgren, E. Russell, C. D. Von Bargen, R. Abel, R. A. Friesner and E. D. Harder: OPLS4: Improving Force Field Accuracy on Challenging Regimes of Chemical Space. *J Chem Theory Comput*, 17(7), 4291-4300 (2021) doi:10.1021/acs.jctc.1c00302

6. R. J. Loncharich, B. R. Brooks and R. W. Pastor: Langevin dynamics of peptides: the frictional dependence of isomerization rates of N-acetylalanyl-N'-methylamide. *Biopolymers*, 32(5), 523-35 (1992) doi:10.1002/bip.360320508

7. N. Gronbech-Jensen and O. Farago: Constant pressure and temperature discrete-time Langevin molecular dynamics. *J Chem Phys*, 141(19), 194108 (2014) doi:10.1063/1.4901303
