# Supplementary material for: AlphaFold predicted structure of the Hsp90-like domains of the neurodegeneration linked protein sacsin reveals key residues for ATPase activity
Source: Front Mol Biosci. 2023 Jan 13;9:1074714. doi: 10.3389/fmolb.2022.1074714 (PMC9880540; doi:10.3389/fmolb.2022.1074714)
Supplement: Supplementary file 2 [file Image1.pdf]

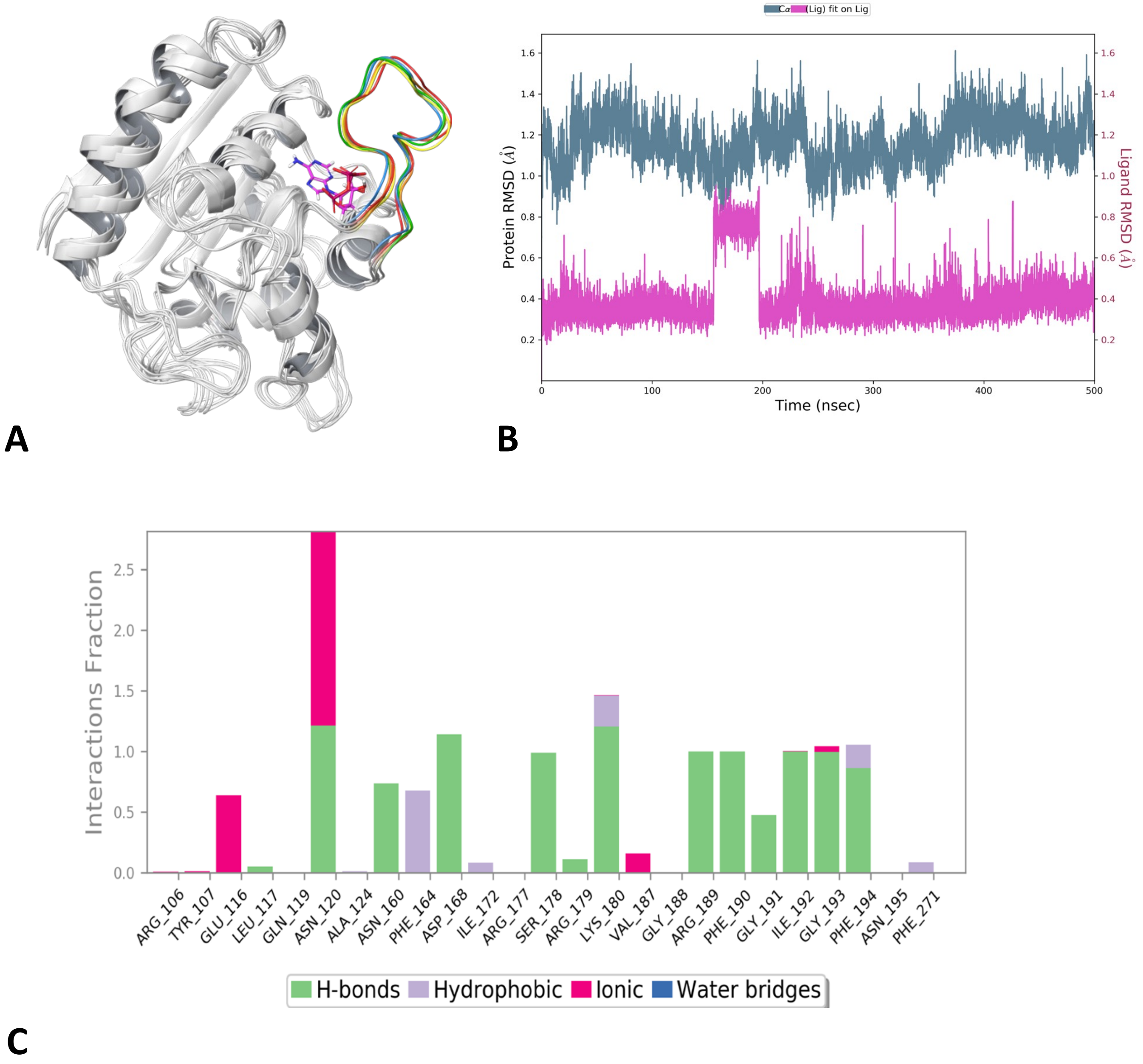

Figure S1. Molecular Dynamic simulation of sacsin-ATP complex. (A) Cluster analysis of the loop; the most populated cluster is shown in red, the second in orange, the third in yellow, the fourth in green and the least populated in blue. **(B)** RMSD analysis of protein C-alpha (blue) and ligand (magenta). **(C)** Protein-ligand interaction diagram. Interactions are categorized into four types: Hydrogen Bonds (green), Hydrophobic (lilac), Ionic (pink) and Water Bridges (blue). The stacked bar charts are normalized over the course of the entire trajectory. Values over 1.0 are possible as some protein residue may make multiple contacts of same subtype with the ligand.

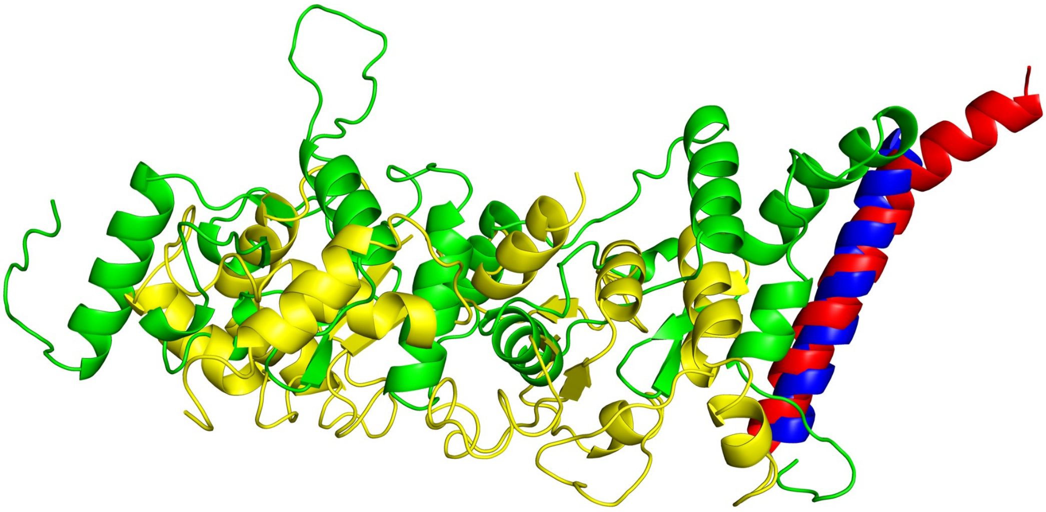

Figure S2. Hsp90 middle and C-terminal regions and corresponding segments of sacsin that do not superimpose. PyMol cartoon of Hsp90 (yellow and blue, residues 386 to 677) and sacsin (green and red, residues 487 to 772). The blue long helix of the Hsp90 middle domain and that of sacsin are superimposed. The rest of the structure shows no immediately recognisable common topology.

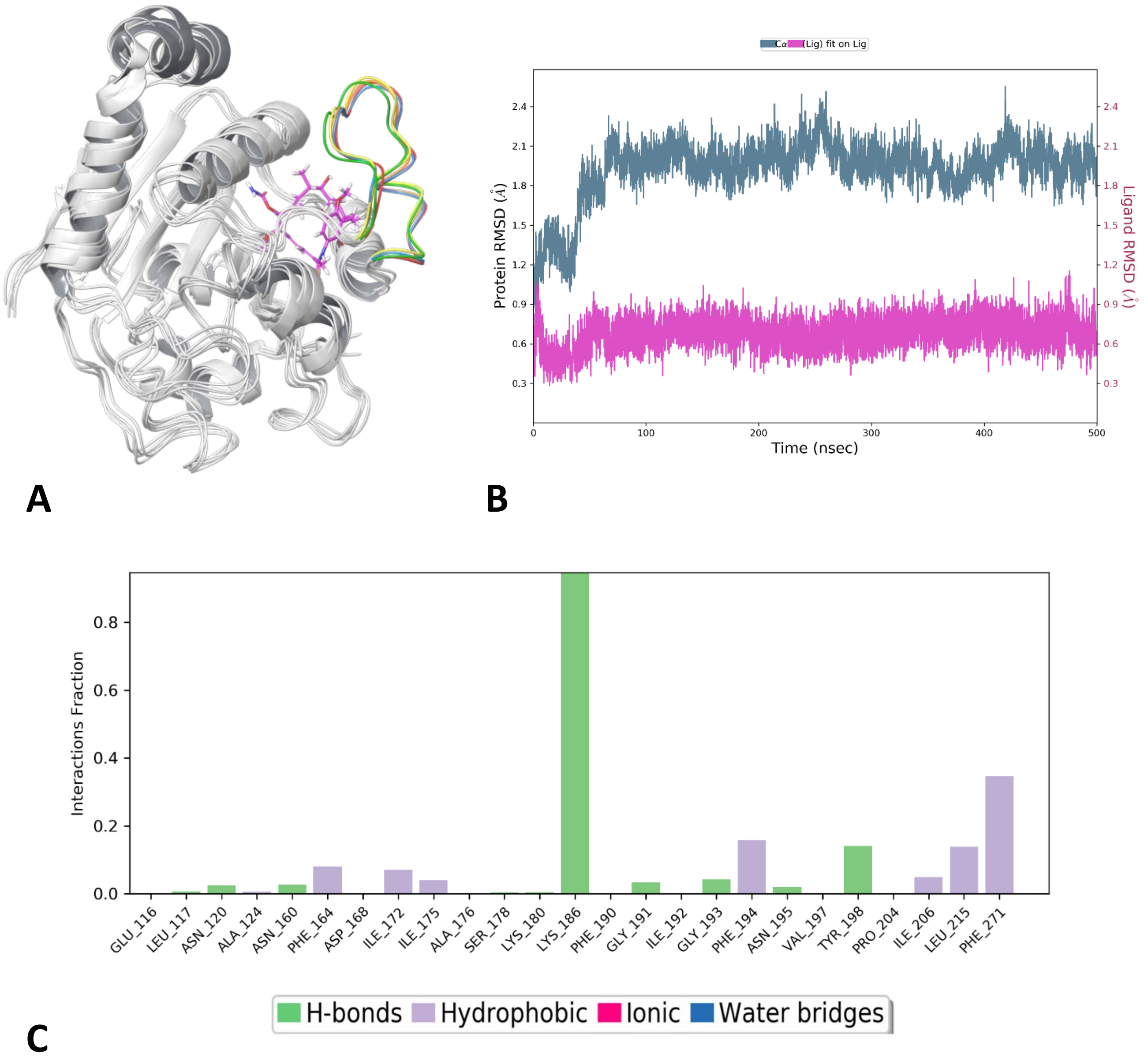

Figure S3. Molecular Dynamic simulation of sacsin-geldanamycin complex. (A) Cluster analysis of the loop; the most populated cluster is shown in red, the second in orange, the third in yellow, the fourth in green and the least populated in blue. **(B)** RMSD analysis of protein C-alpha (blue) and ligand (magenta). **(C)** Protein-ligand interaction diagram. Interactions are categorized into four types: Hydrogen Bonds (green), Hydrophobic (lilac), Ionic (pink) and Water Bridges (blue). The stacked bar charts are normalized over the course of the entire trajectory. Values over 1.0 are possible as some protein residue may make multiple contacts of same subtype with the ligand.

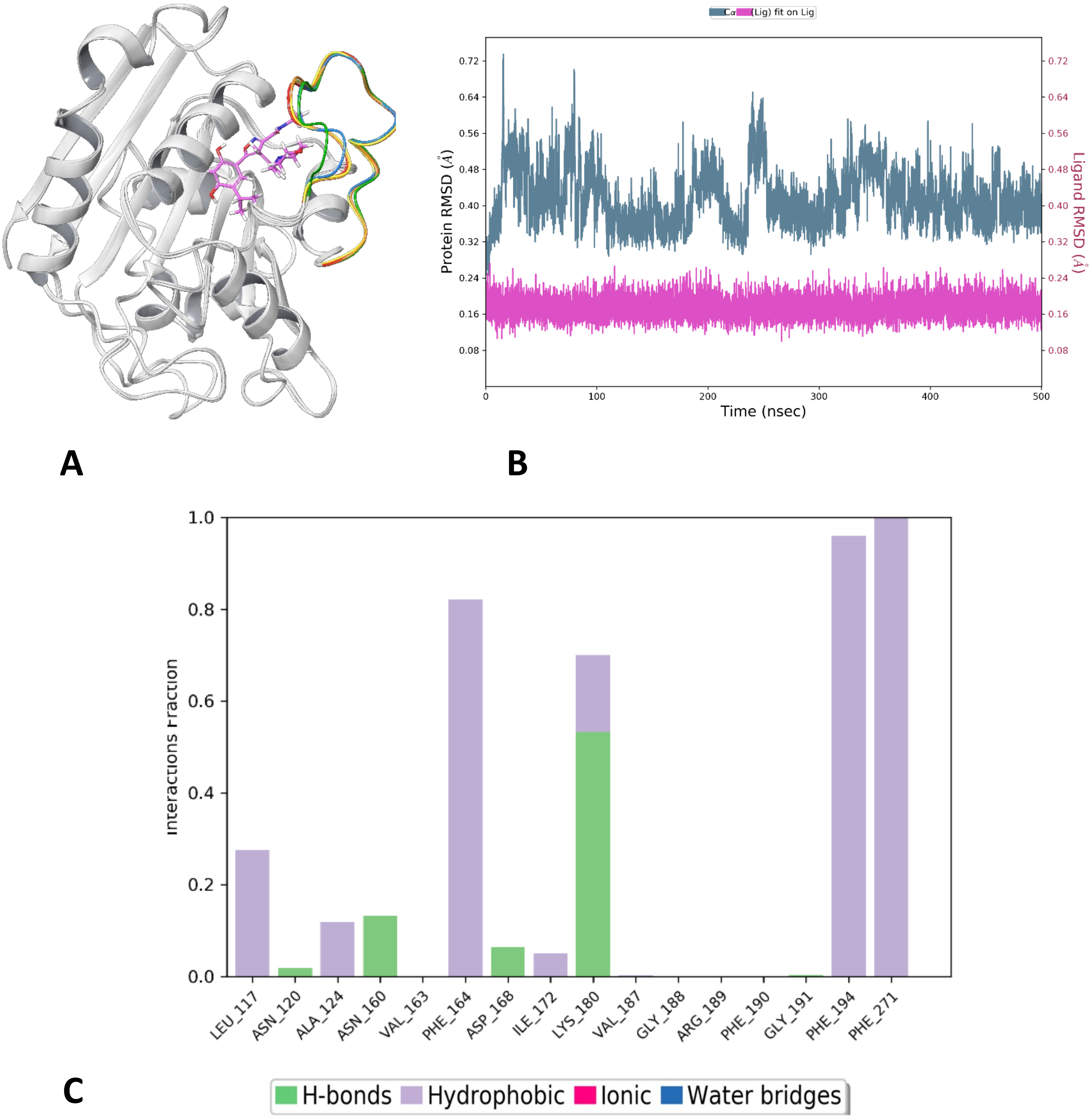

Figure S4. Molecular Dynamic simulation of sacsin-AUY922 complex. (A) Cluster analysis of the loop; themost populated cluster is shown in red, the second in orange, the third in yellow, the fourth in green and the least in blue. **(B)** RMSD analysis of protein C-alpha (blue) and ligand (magenta). **(C)** Protein-ligand interaction diagram. Interactions are categorized into four types: Hydrogen Bonds (green), Hydrophobic (lilac), Ionic (pink) and Water Bridges (blue). The stacked bar charts are normalized over the course of the entire trajectory. Values over 1.0 are possible as some protein residue may make multiple contacts of same subtype with the ligand.
